# Empirical validation of integrated stock assessment models to ensuring risk equivalence: A pathway to resilient fisheries management

**DOI:** 10.1371/journal.pone.0302576

**Published:** 2024-07-02

**Authors:** Laurence T. Kell, Iago Mosqueira, Henning Winker, Rishi Sharma, Toshihide Kitakado, Massimiliano Cardinale

**Affiliations:** 1 Centre for Environmental Policy, Imperial College London, London, United Kingdom; 2 Wageningen Marine Research, IJmuiden, The Netherlands; 3 Department of Aquatic Resources, Institute of Marine Research, Swedish University of Agricultural Sciences, Lysekil, Sweden; 4 Fishery and Aquaculture Policy and Resources Division, Food and Agricultural Organization, Rome, Lazio, Italy; 5 Department of Marine Biosciences, Tokyo University of Marine Science and Technology, Minato, Tokyo, Japan; Aristotle University of Thessaloniki, GREECE

## Abstract

The Precautionary Approach to Fisheries Management requires an assessment of the impact of uncertainty on the risk of achieving management objectives. However, the main quantities, such as spawning stock biomass (SSB) and fish mortality (F), used in management metrics cannot be directly observed. This requires the use of models to provide guidance, for which there are three paradigms: the best assessment, model ensemble, and Management Strategy Evaluation (MSE). It is important to validate the models used to provide advice. In this study, we demonstrate how stock assessment models can be validated using a diagnostic toolbox, with a specific focus on prediction skill. Prediction skill measures the precision of a predicted value, which is unknown to the model, in relation to its observed value. By evaluating the accuracy of model predictions against observed data, prediction skill establishes an objective framework for accepting or rejecting model hypotheses, as well as for assigning weights to models within an ensemble. Our analysis uncovers the limitations of traditional stock assessment methods. Through the quantification of uncertainties and the integration of multiple models, our objective is to improve the reliability of management advice considering the complex interplay of factors that influence the dynamics of fish stocks.

## Introduction

The main objectives of fisheries management are to ensure that stocks provide the maximum sustainable yield (*MSY*) and are maintained with high probability above a point where productivity is impaired. Therefore, the provision of fisheries management advice requires the assessment of the state of the stock relative to target and limit reference points, the prediction of the response of the stock to management, and the verification that the predictions are consistent with observations. However, the main quantities of interest, spawning stock biomass (*SSB*) and fishing mortality (*F*), cannot be observed. Therefore, models with latent variables are required to assess the state of the stock, derive reference points, and propose management actions.

Currently, the primary diagnostics used to select and reject assessment models are to examine residuals to verify goodness of fit and to perform a retrospective analysis to verify stability. However, residual patterns can be removed by adding more parameters than justified by the data, and retrospective patterns by ignoring the data [1]. Validation using empirical data plays an important role in sustainability science [2], and models must be validated if they are to provide robust and credible advice [3]. This requires assessing whether it is plausible that a system equivalent to the model generated the data [4] and whether assumptions are violated. Therefore, an alternative to residual and retrospective analysis is to perform a hindcast by omitting recent observations and then predicting their out-of-sample values [5]. Prediction skill is a measure of the precision of a predicted value unknown to the model relative to its observed value. Prediction skill can be used to explore model misspecification and data conflicts, to help identify alternative hypotheses, and can be used as objective methods to select, reject, and assign weights to models [6].

To ensure that advice on the consequences of tactical and strategic management actions is robust, the Precautionary Approach to Fisheries Management [7] requires the quantification of uncertainty and reduces the risk that uncertainty hinders the achievement of management objectives. Ideally, risk equivalence should be considered so that objective-based management decisions can be maintained within acceptable risk levels and deliver results consistent with expectations and trade-offs between them [8]. In the context of single species advice, this means that in situations with poor or limited data and consequently greater uncertainty, management should not allow greater risks as required in tiered assessment frameworks [9].

There are three main modelling paradigms to provide advice: the best assessment, the model ensemble, and Management Strategy Evaluation (MSE). Each has its own means of determining quality that implies plausibility, but plausibility is rarely objectively defined. In the best-assessment paradigm, alternative models are fitted to historical time series of independent and fishery-dependent data, and then a single scenario is selected based on goodness-of-fit diagnostics [10]. However, there is often a lack of information in stock assessment data on system processes [11–14], and the data sets may conflict. Therefore, ensembles in which model estimates are combined, consisting of as few as two [15] or thousands [16] of models, may be preferred. The third paradigm, MSE, is a formal way of simulation-testing feedback control [17]. The aim is to design robust and fault-tolerant control systems that allow management objectives to be met despite the uncertainty represented by Operating Models that represent resource dynamics [18]. In MSE, advice may be provided by an empirical control rule using an indicator based on data rather than a stock assessment. The indicator should be able to track the status or trends in the stock, and after implementation a review should be performed to evaluate whether management objectives have been achieved. Therefore, prediction skill is valuable for selecting Operating Models which may be conditioned on a stock assessment, the selection of indicators, and in assessments conducted as part of implementation reviews.

An assumption under the best assessment and ensemble paradigms is that model outputs quantify the consequences of the uncertainties in model inputs. To do this requires an uncertainty analysis, for example, in the best assessment, sampling the values of fixed parameters from prespecified distributions, or for an ensemble by including all plausible models. In the latter case, multiple models are run for scenarios related to alternative model structures and values for prespecified parameters, and the results are combined to provide advice [19]. [20] used a set of southern bluefin assessment scenarios to cover the range of interpretations of the main uncertainties. However, multiple alternative model structures may be equally plausible and therefore the number of model scenarios required to perform a full uncertainty analysis may be infeasibly large. Instead, sensitivity analyses are generally preferred, i.e. systematic investigation of the reaction of model outputs to extreme values of the model inputs and drastic changes in the model structure. It is possible to perform a sensitivity analysis of the model around a reference case and then use it as part of a first-order uncertainty analysis [21]. In any case, an objective approach should be used for selecting, screening, and weighting hypotheses, to overcome artefacts and biases introduced by a “cherry picking” approach [22]. In particular, since divergent views and beliefs mean that uncertainties can be used to support stakeholder positions and to strengthen or weaken management measures [23].

Providing probabilistic advice involves determining the uncertainty of the model output derived from uncertain inputs and assumptions of the model [24]. Increasingly, multiple models are used to develop advice [19, 25], either combined to make probability statements or as Operating Models in MSE to represent alternative hypotheses reflecting uncertainty about resource dynamics to which management should be robust. Multiple models may be combined using an ensemble that treats each model scenario as an alternative hypothesis and implicitly recognises that each may explain the data equally well or be weighted based on an estimate of plausibility [26, 27]. In the case of the best assessment, uncertainty is based on confidence or credible intervals, whereas in an ensemble estimates are combined across models. Although ensembles can improve model predictions, they must themselves be validated, formed from a diverse set of models to minimise redundancy, and built on a data set representative of the population to which they are applied [28]. In MSE, a set of references or a single reference case is developed. These scenarios are a limited set of Operating Model scenarios that include the most significant uncertainties in the structure, parameters, and data of the model. Alternative scenarios should be highly plausible and have a significant impact on the performance statistics of candidate MPs. In addition, a robustness set should be developed to assess performance across a wider range of plausible scenarios. These should represent hypotheses that are less plausible than those in the reference set and focus on challenging circumstances with potentially negative consequences that should be avoided.

In all paradigms, plausibility is rarely objectively defined. Therefore, we first explore the impact of uncertainty and then demonstrate ways to define the plausibility of alternative models by evaluating criteria based on retrospective bias and prediction skill [1]. We then compare model weighting schemes and discuss how the process can be generalised.

## Material and methods

The choice of scenarios for assessment models and methods of estimating uncertainty has an impact on the risk of exceeding the limit and missing target reference points. The procedure for selecting and rejecting scenarios in all paradigms will determine the advice. To better understand the impact of uncertainty on stock assessment advice and the risk of not meeting conservation and sustainability objectives, we used the uncertainty grid developed by the Indian Ocean Tuna Commission (IOTC) for albacore tuna (*Thunnus alalunga*) as an example.

**Fig 1:**
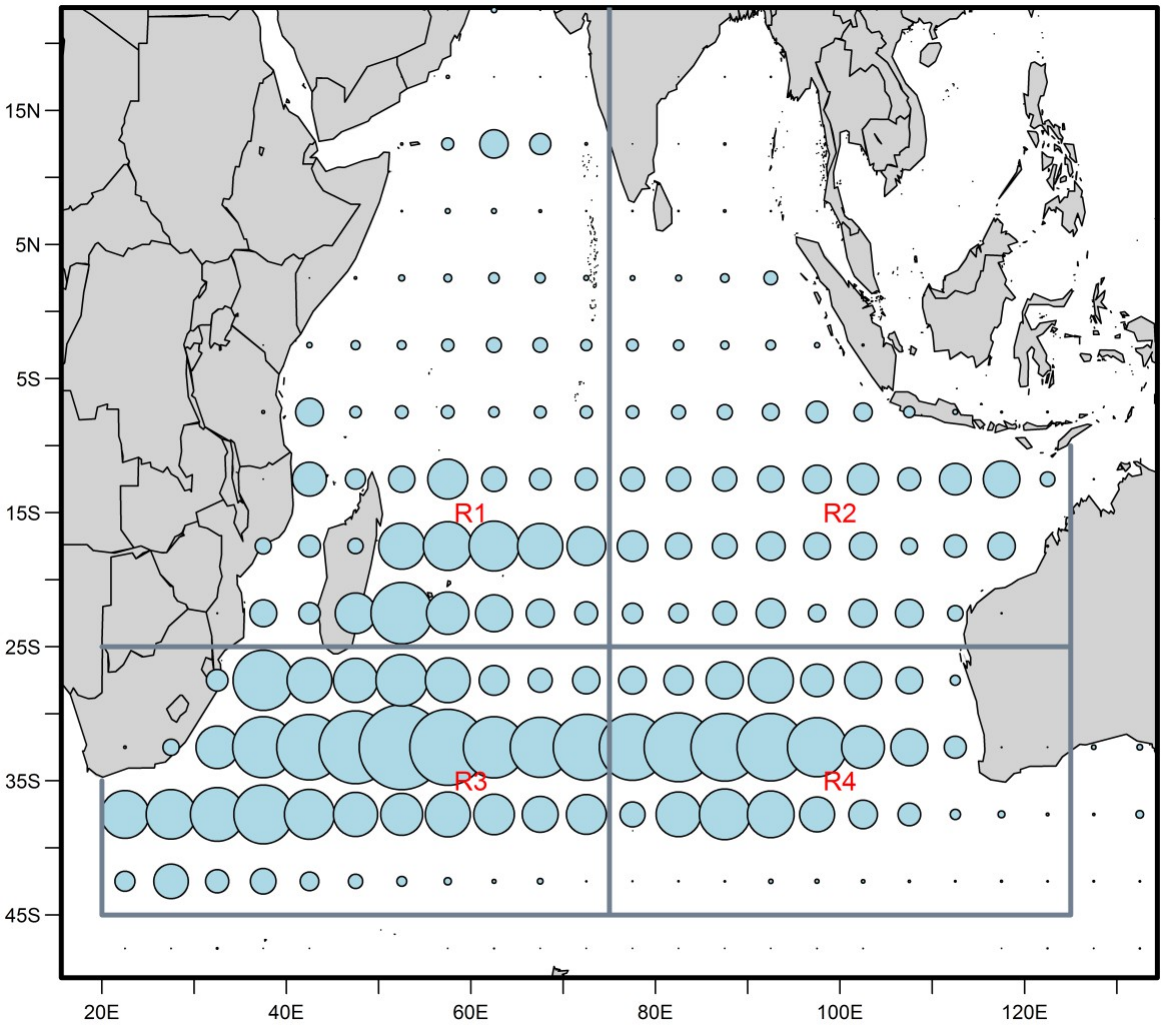
Relative distribution of Indian Ocean albacore tuna catches by assessment region.

The data set for the Indian Ocean albacore assessment includes records of catches and landings, abundance indices based on catch per unit of effort (CPUE) and samples of length composition. The assessment partitions the Indian Ocean into four regions, divided latitudinally along the 25°S parallel and longitudinally along the 75°E meridian. Fig 1 shows the distribution of catches between the four regions. The assessment includes 11 fisheries, including an aggregated longline fishery for each region [29], and a set of standardised CPUE indices that have been derived from the longline catch and effort data provided by Japan, Korea, and Taiwan, China [30]. Area 3 is considered to represent the core of the distribution of the stock.

### Uncertainty grid

Tuna Regional Fisheries Management Organisations commonly develop uncertainty grids to condition models in integrated stock assessments to account for uncertainties in parameters that cannot be estimated from the data, and data conflicts [19, 31–36]. Grids consist of different plausible combinations of assumptions, fixed parameter values, and data sets. However, it is not always clear whether this is intended as an uncertainty analysis or a sensitivity analysis.

The uncertainty grid developed by the IOTC for albacore tuna (*Thunnus alalunga*), is a full factorial design with 1,440 model configurations [37] (Table 1). This is sufficient to provide contrast, but not too large to be unmanageable. We used Bayesian Markov Chain Monte Carlo (MCMC) methods [38] to estimate the uncertainty of the parameters for the base case. The uncertainty grid includes multiple configurations of integrated assessment models based on current best-knowledge and available data [39]. The grid was conditioned using stock syntheses [40].

**Table 1:** Operating Model scenarios; reference case values in bold.

Key	Factor	Levels (N)	∏ N	Values
M	Natural mortality (M, juveniles/adults)	5	5	0.2, **0.3**, 0.4, 0.4/0.3, 0.4/0.2
h	Steepness of the stock-recruitment relationship	3	15	**0.7**, 0.8, 0.9
SigmaR	Variability of recruitment (sigmaR)	2	30	**0.4**, 0.6
ESS	Effective Sampling Size of the length composition data (ESS)	3	90	20, **50**, 100
CV	CV for fit to CPUE (cpuecv)	4	360	0.2, **0.3**, 0.4, 0.5
q	Yearly increase in catchability coefficient of CPUE (llq)	2	720	**0%**, 1%
sel	Selectivity (llsel)	2	1440	**logistic**, double normal

In the Indian Ocean albacore stock case, several factors limit the ability to obtain robust model fits. These include problems with data completeness and quality [41], not limited to but including total catch statistics, length distribution in catches, and biological information. Therefore, a full factorial design of alternative model configurations based on parameter choices for which there is insufficient information in the data to estimate them or to decide between alternative options was used to construct the uncertainty grid (Table 1).

The reference case, considered by the IOTC the most plausible among a set of candidate models, was extended by selecting alternative values for fixed-parameter values and data weighting to develop the grid. Factors include i) alternative values of natural mortality (M) for juveniles (ages 0 to 4) and adults (age 5 or older); ii) two values for recruitment variability (sigmaR) of 0.4 and 0.6; iii) three values for the steepness (*h*) of the stock-recruitment relationship 0.7, 0.8, and 0.9; iv) four values for the coefficient of variation in the CPUE series of 0.2, 0.3, 0.4 and 0.5; v) three values for the relative weight of length sampling data in the total likelihood through changes in the effective sampling size parameter, 20, 50 and 100; vi) two scenarios for the effective catchability of the CPUE fleet: It was assumed that the fleet had not improved catchability plus an alternative scenario that considered a 1.0% yearly increase; vii) two possible functional forms for the long-line fleet selectivity were considered: a logistic function (Log), where selectivity stays at the maximum level for older sizes, or a double normal (DoNorm), where selectivity decreases for larger sizes. This resulted in a grid of 1,440 individual models, which covers most, but not all, plausible sources of uncertainty.

#### Model estimates

Provision of fisheries management advice requires the assessment of stock status relative to reference points to prevent growth, recruitment, economic and target overfishing. Growth and recruitment overfishing are generally associated with threshold or limit reference points, while economic overfishing can be expressed in terms of targets or limits [42]. The difference between targets and limits is that indicators may fluctuate around targets, but in general limits should not be crossed. Target overfishing occurs when a target is overshot, while variations around a target are not necessarily considered serious unless a consistent over or undershoot becomes apparent. In contrast, even a low probability of violating a limit reference point (LRP) may indicate the need for immediate action. *F*_*MSY*_ is often considered a limit and thresholds or triggers can also be implemented to initiate a management action.

Patterns or fluctuations generated by a model have an impact on advice [43]. Therefore, two key properties of the output of the assessment model are the production function and the process error. The former is used to calculate analytical reference points [44], while the latter is a source of additional variability that is not represented by the main structure of the model [45]. Process error may be due to variations in biotic or abiotic processes; that is, the drivers of population fluctuations that ecologists are interested in quantifying [46]. Process errors arise when a deterministic component of a population model incorrectly describes population processes. Such process variation can be found, for example, in recruitment, fishery selectivity, or sampling processes [47].

If the stock size is represented by biomass and changes by the time-accounting equation [48]

$$\begin{array}{c} log\left(B_{t+1}\right)=log(B_{t}-C_{t}+P\left(B_{t}\right) \end{array}$$ (1)

where *B* is exploitable biomass and *C* is catch, then surplus production (*P*(*B*_*t*_)) is the net change in biomass if *C* = 0, and represents the net addition to biomass due to the recruitment of fish too small to be taken into account in *B*, plus growth minus loss of natural mortality.

In integrated stock assessment models, the process error *ϵ*_*t*_ can be estimated as the difference between the deterministic expectation and the stochastic realisation for biomass *B*_*t*+1_, i.e.

$$\begin{array}{c} \epsilon_{t}=log(\frac{B_{t}-C_{t}+Pt}{B_{t+1}}) \end{array}$$ (2)

Full details of the methods in the next section are provided in Supporting Information.

#### Goodness of fit

Goodness of fit involves assessing the fit of the model through the examination of residual patterns to identify any systematic misfits, such as bias or trends, that could indicate misspecification of the model. Simple residual plots are used along with statistical tests, including the Runs test [49], to detect deviations from expected patterns. Information-theoretic criteria, such as Akaike’s Information Criterion (AIC) and its variants (AICc for small sample sizes), [50], GIC [51], DIC [52] and WAIC [53]), are often applied to select models that best balance fit and complexity, considering both frequentist and Bayesian approaches. However, since the scenarios included different weightings due to data conflicts, Information-theoretic criteria cannot be used for weighting in such cases.

#### Model consistency

We evaluated model consistency through retrospective analysis, specifically using Mohn’s rho (*rho*_*M*_), to measure systematic errors over time as data are sequentially omitted from the analysis [54]. This approach helps identify biases that could affect management decisions.

#### Model validation

Validation focused on the model’s ability to predict unseen data, employing hindcasting in which data points are removed using a tail cutting approach, i.e. removing data sequentially from the most recent years backward. and predicted by the model [1]. This was primarily applied to catch-per-unit-effort (CPUE) data due to limitations in data availability, especially from regions beyond national jurisdiction. The prediction skill of the model was quantified using the Mean Absolute Scaled Error (MASE) [55], comparing the model forecasts with a naïve forecast over specified forecast horizons. MASE values less than 1 indicate predictions more accurate than the naïve forecast, offering a clear criterion to evaluate model performance.

The Diebold-Mariano test can be applied to compare the predictive precision of our model against a naive benchmark, providing a statistical basis to evaluate the significance of differences in performance prediction [56].

## Results

### Time series analysis

**Fig 2:**
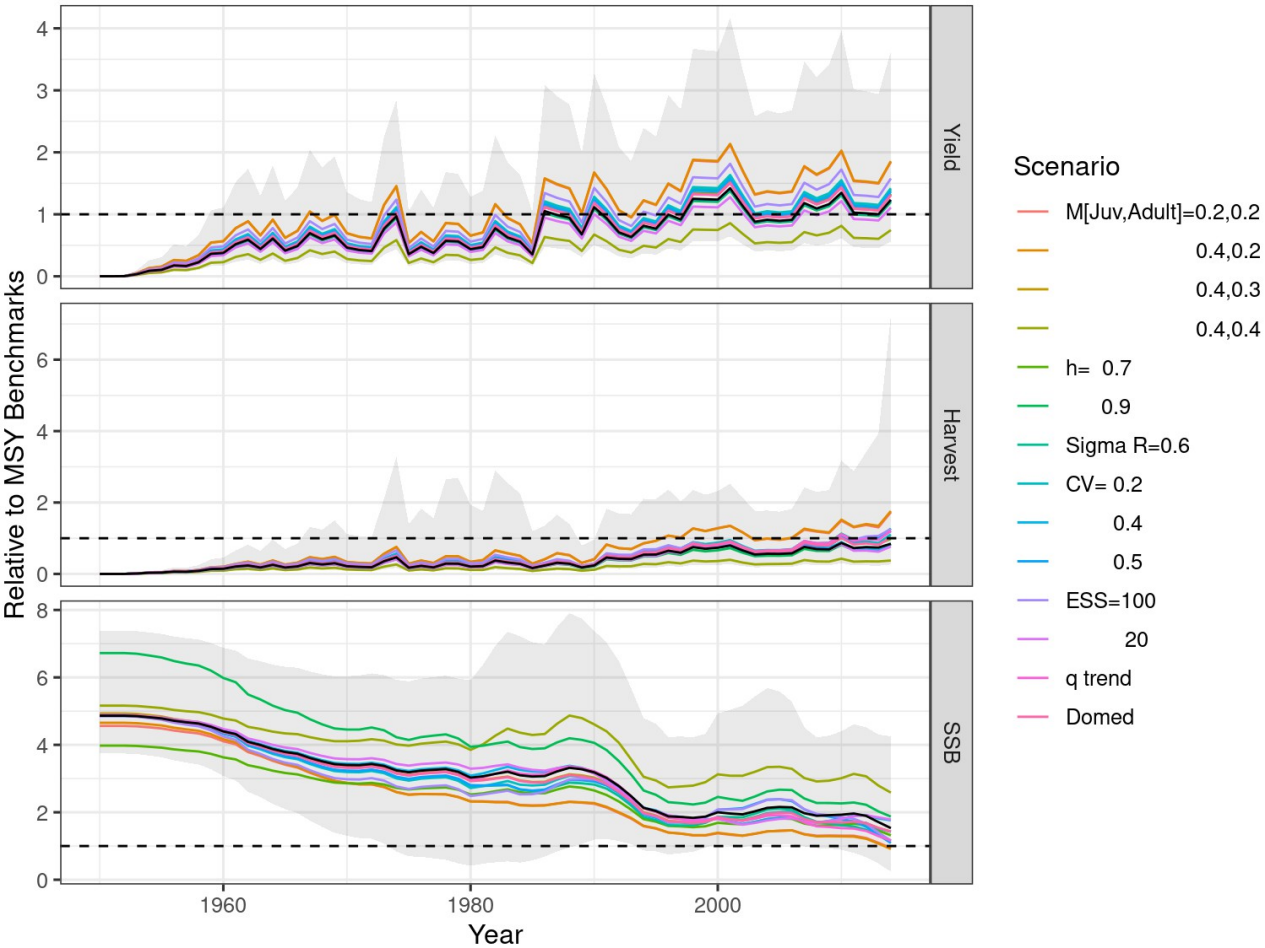
Time series of yield, harvest rate, and spawning stock biomass, relative to *MSY* reference points, for the main effects in the uncertainty grid; thick black line is the base case.

Time series of the yield, fishing mortality, and SSB relative to their corresponding MSY reference points is summarised in Fig 2. Absolute estimates of biomass and fishing mortality are uncertain because M is not well known and the use of relative values allows focus to be on trends and proportional changes. The reference case (black line) and the main effects where the levels of each factor vary one by one are shown; the ribbons delimit the range across all 1440 scenarios. The base case trajectories are in the middle of the range, as the scenarios were based on varying factors around the base case. Trajectories exhibit similar variability within a quantity, but tend not to intersect as they change at similar rates. Catches vary the most reflecting the impact of operational and environmental factors, while SSB the least as it is a modelled quantity and process error is accounted for in recruitment and selectivity. SSB estimates decreased while harvest rate and catches increased, that is, they are inversely correlated, as a large stock with low exploitation or vice versa can explain the observed catches. The yield, which represents the recorded catch, exhibits significant interannual variations, and since 2000, the catches have been above or close to MSY. Estimates are sensitive to the assumed level of M, since the scenarios for M = 0.2 and 0.4 bracket the other main scenarios. SSB remains above *B*_*MSY*_, but shows a downward trend. The harvest rate follows a trend similar to the yield but with less variability. For most time series, the harvest rate remains below *F*_*MSY*_. In recent years, some scenarios have shown that harvest rates reach or exceed *F*_*MSY*_. SSB scenarios were based on, but remain above, *B*_*MSY*_.

### Stock status

**Fig 3:**
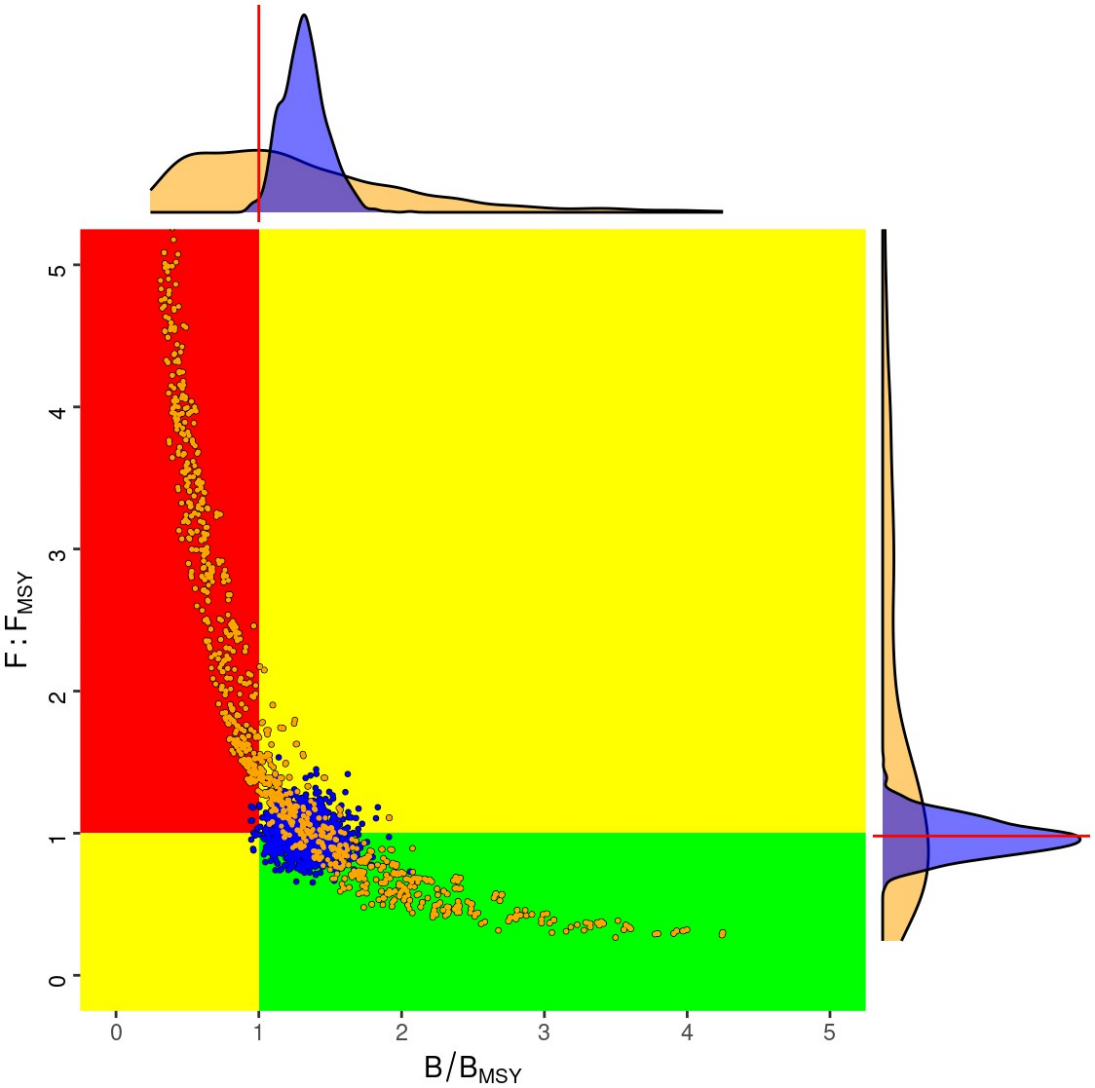
Kobe phase plot showing spawning biomass and fishing mortality, relative to *MSY* target reference points. Yellow points correspond to all the deterministic model estimates in the uncertainty grid, and blue points to the MCMC posteriors for the base case.

The status of the stock in the terminal year for all 1440 scenarios is summarised in a Kobe phase plot (Fig 3). The green quadrant provides an assessment of sustainability, as it indicates a well-managed fishery where *SSB*/*B*_*MSY*_ > 1 and (*F*/*F*_*MSY*_ < 1). The red zone shows where a stock is overfished and where overfishing occurs. Two sets of data points are plotted: mustard points for the 1440 deterministic model estimates within the uncertainty grid and blue points for the MCMC base case posteriors, which account for uncertainty in the model parameters. Marginal distributions, depicted along the plot’s axes, allow probabilities from model estimates and the MCMC analysis to be compared, i.e. targets by the central tendency and limits by the tails. The Kobe phase plot reveals a tendency for the deterministic model estimates to fall within the red zone, indicating overexploitation. However, the MCMC posteriors cluster toward the green zone, suggesting a more sustainable stock status. The contrast highlights the role of uncertainty in stock evaluations and decision making.

**Fig 4:**
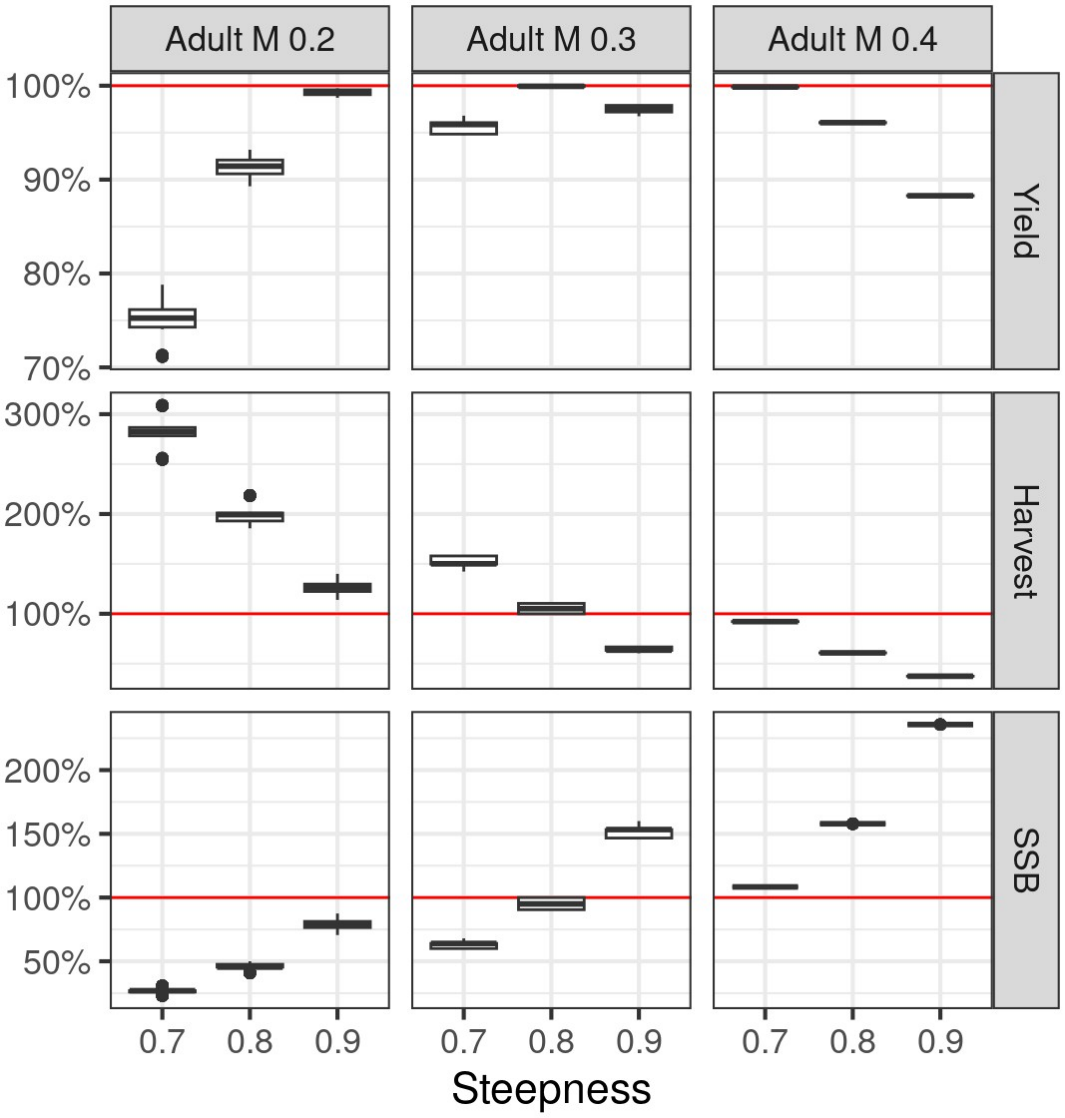
Deterministic estimates in the final year from the uncertainty grid of biomass, harvest rate, and yield relative to *MSY* reference points; summarised by adult M and steepness.

The current yield, F, and SSB relative to their MSY benchmarks are summarised in Fig 4) by natural mortality and steepness. The ratios are derived from the uncertainty grid, which also considers, but is not significantly influenced, factors such as juvenile M, ESS, CPUE CV, catchability, and selectivity. Therefore, these additional factors are integrated into the box and whisker plots. There is a compensatory relationship between steepness and M, since high steepness and low M result in outcomes comparable to those with low steepness and high M. The figure further illustrates that an increase in M is correlated with a decrease in *F*/*F*_*MSY*_. A relationship that has implications for data-deficient situations where M is used as a substitute for *F*_*MSY*_. As seen in the Kobe Phase plot, there is an inverse relationship between *SSB*/*B*_*MSY*_ and *F*/*F*_*MSY*_; scenarios with higher values of M and steepness are associated with lower fishing mortality ratios and higher biomass ratios of the spawning stock relative to MSY. This suggests that for model configurations with higher M and steepness, the stock is less exploited and has healthier spawning biomass compared to the MSY benchmarks.

### Production functions

**Fig 5:**
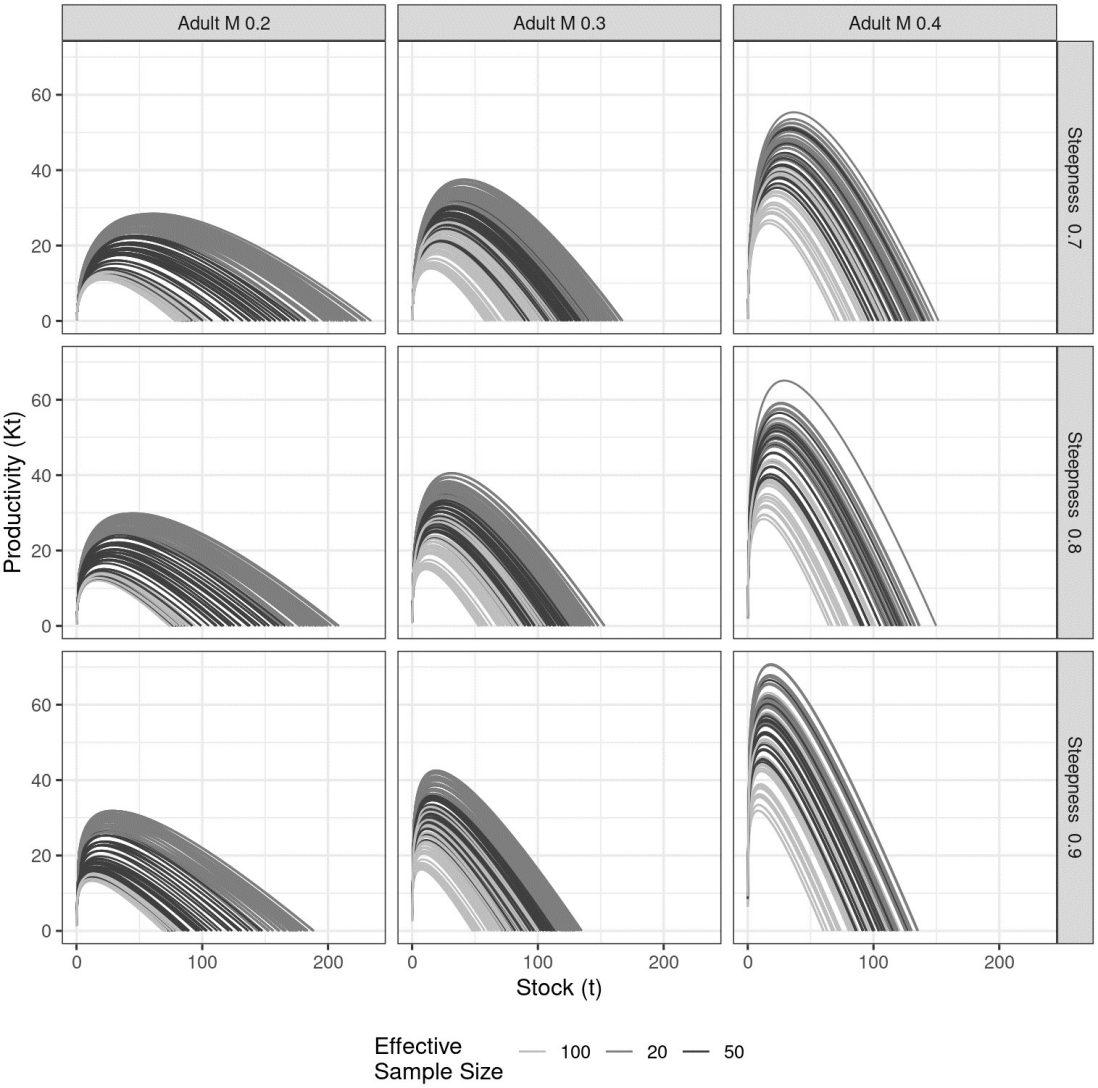
Plots of equilibrium yield against equilibrium biomass (i.e. the production functions) by natural mortality of adults (column), steepness (row), and effective sample size (shading).

Plots of the relationship between equilibrium yield and equilibrium biomass, which are used to derive MSY benchmarks, are commonly called production functions. To understand the impact of the uncertainty grid on the production function, these are summarised in Fig 5 by the natural mortality rates of adults (M) and the steepness of the stock-recruitment relationship. The shading within the plots indicates the effective sample size, a measure of the amount of size information available to estimate the status of the stock. The shape and peak of these curves vary with the natural mortality rate and steepness, illustrating how sensitive the fishery’s productivity is to these key life history parameters. Again, M and steepness have a large effect; increasing adult *M* results in higher productivity, and therefore *MSY*, while increasing steepness shifts the curve to the left, increasing *F*_*MSY*_ (since *F* is equivalent to catch/biomass), making the stock more resilient to fishing pressure.

### Process error

**Fig 6:**
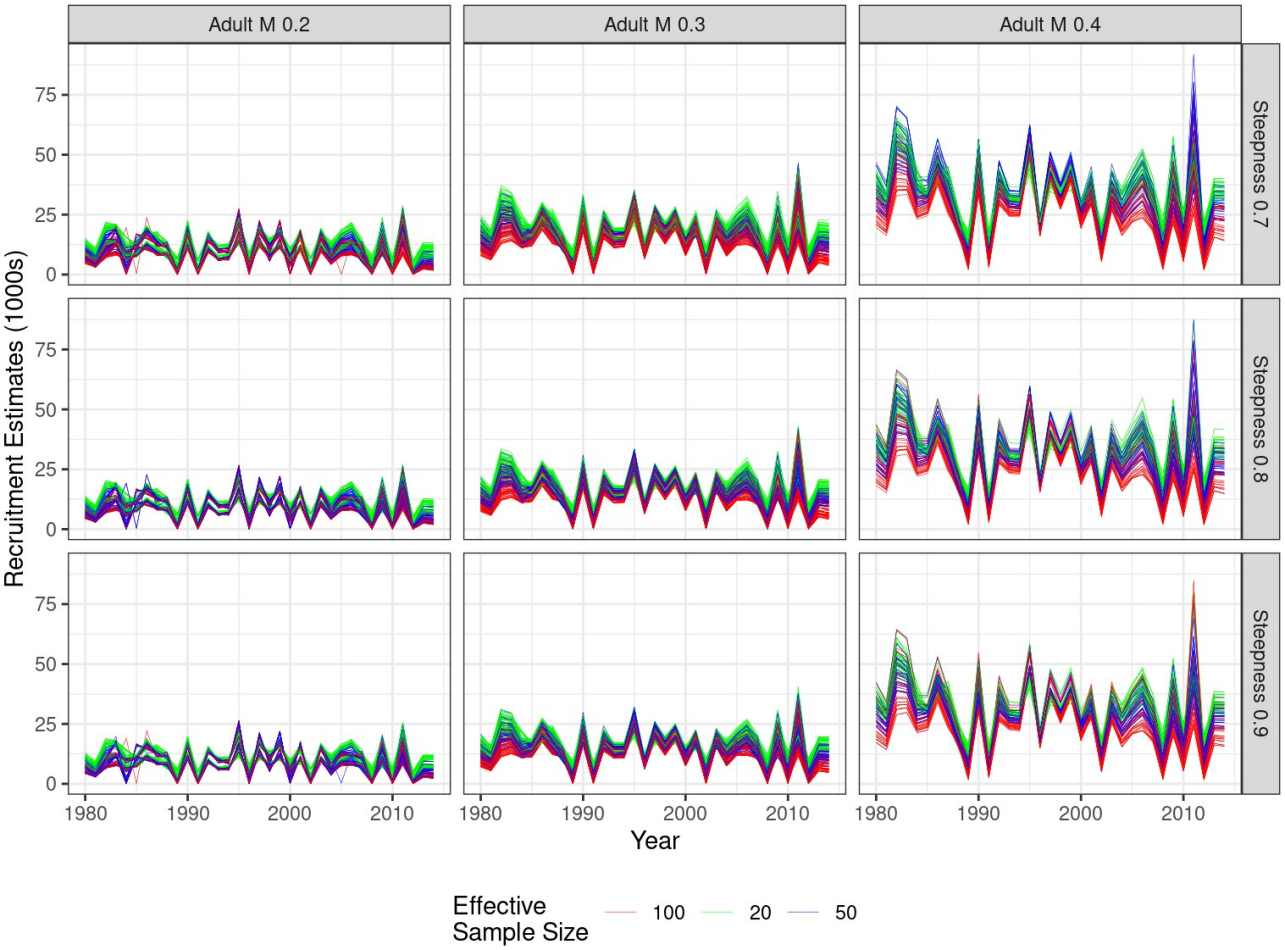
Time series of recruitment estimates by natural mortality of adults (column), steepness (row) and effective sample size (shading).

**Fig 7:**
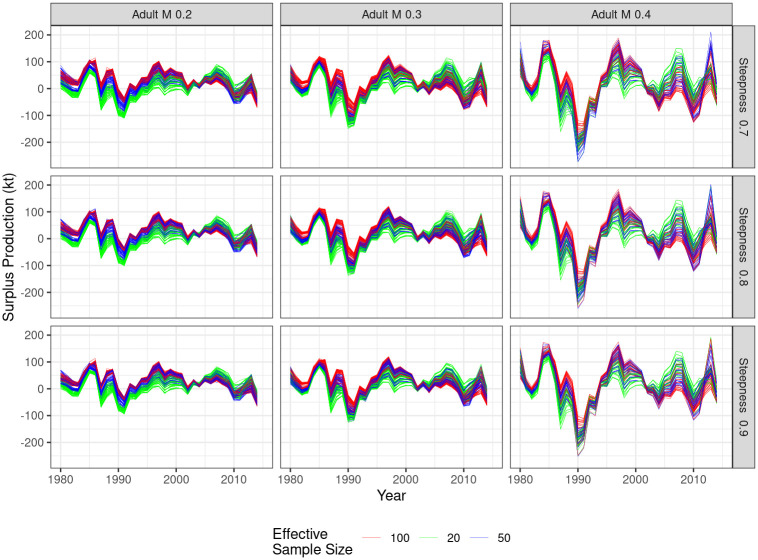
Time series of estimates of surplus production, i.e. *B*_*y*+1_ − *B*_*y*_ + *Cy*, by natural mortality of adults (column), steepness (row) and effective sample size (shading).

A property of the model estimates is process error, modelled in this case by recruitment deviates. Fig 6 show the recruitment deviates and Fig 7 process error. M has the greatest effect, since if adult M is large, then recruitment and variability in the strength of the year class have a great effect on biomass. Steepness and other factors have less of an impact.

### Decision tree analysis

**Fig 8:**
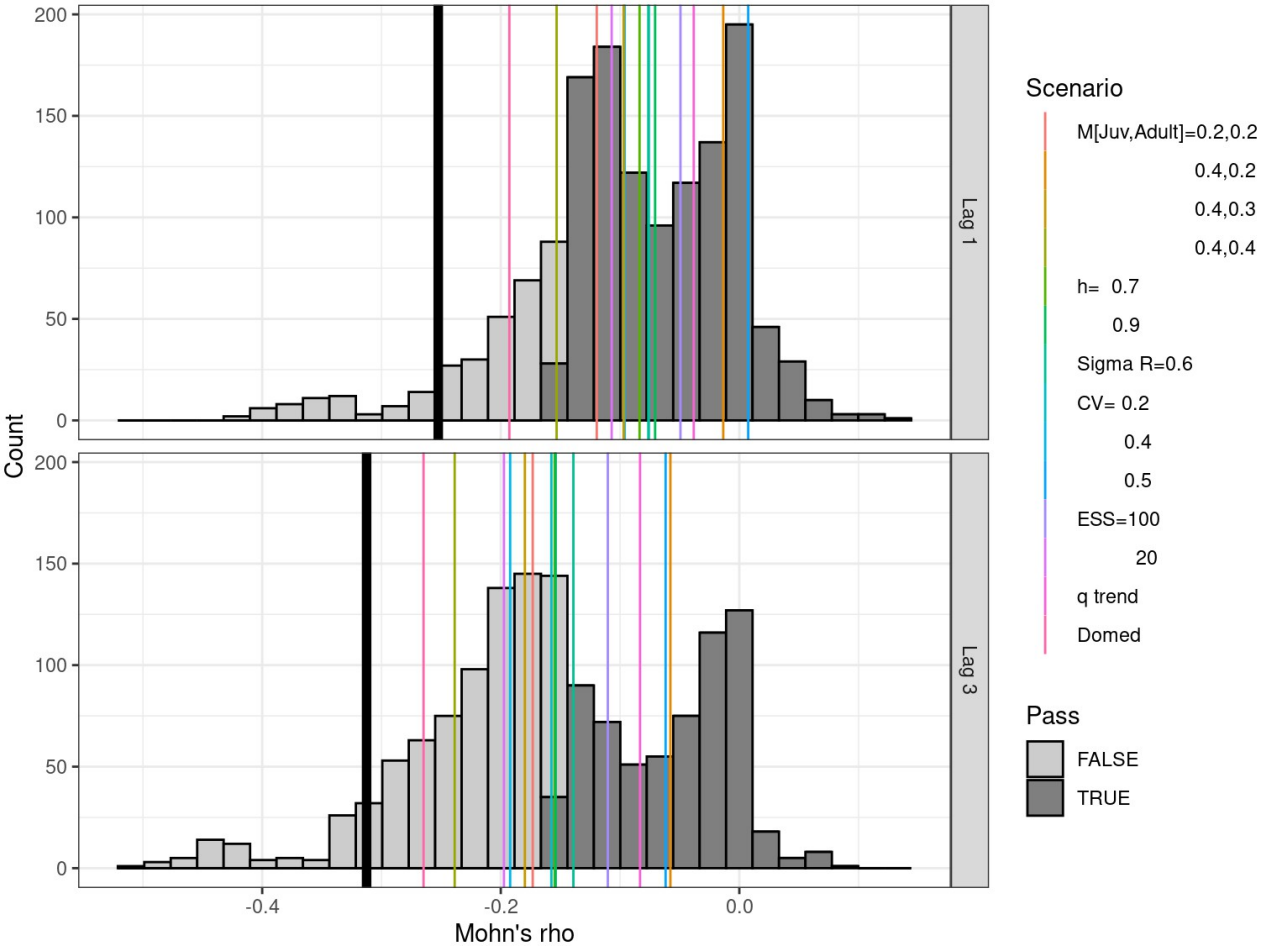
Summary of Mohn’s *ρ* for the uncertainty grid, main effects are indicated by the vertical lines, and pass criteria is when −0.15 < *ρ* < 0.2.

**Fig 9:**
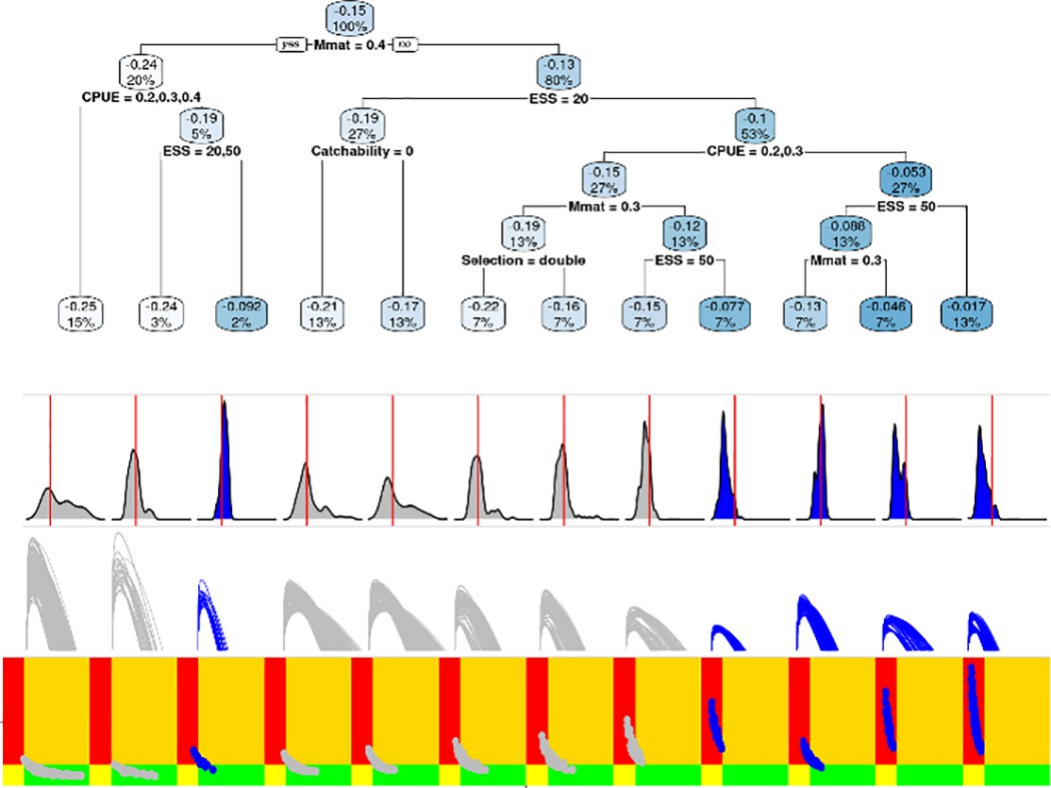
Regression tree identifying the inputs, i.e. grid factors and levels, that influence Mohn’s *ρ* for the 3-step ahead procedure. Below the regression tree are summaries by cluster of MASE (red vertical line is MASE = 1), production functions, and Kobe phase plots.

The retrospective analysis using Mohn’s *ρ* is summarised in Fig 8. The black line identifies the reference case, and the main effects are indicated by the coloured lines. The base case fails with the lowest score, and the scenarios with the lowest retrospective bias are for M 0.4 and 0.2 and CPUE with a CV of 50%. The impact of factors and levels of the uncertainty grid is explored using a regression tree in Fig 9 using Mohn’s *ρ* as response variable. Below the regression tree, the clusters are summarised by their MASE values, production functions, and Kobe plots. Where Mohn’s rho is greater than a value of -0.15 within a cluster, the values are indicated by blue.

The key factors that influence Mohn’s rho are ESS, CPUE CV, catchability, and adult M. The analysis did not select Sigma R, steepness, or selection pattern as influential factors. Therefore, adult M and the relative weights given to the CPUE and the length composition data have the main impact. First clusters are summarised by their MASE values, with a red vertical line indicating a MASE value of 1. MASE is a measure of forecast accuracy, with values less than 1 indicating good predictive performance. Clusters with a median MASE less than 1 are considered to have passed the test, suggesting both stability in assessment and prediction skill. Next, we have the production functions, with those that pass Mohn’s rho test showing the lowest values for MSY, *B*_*MSY*_ and the carrying capacity (K). The last row shows the Kobe phase plots, where the clusters that pass the Mohn’s *ρ* test tend to be those where overfishing is occurring and a stock is overexploited.

## Discussion

The Kobe Phase plot in Fig 3 compared the estimation error of the base case with the model error of the uncertainty grid. The blue points, which denote the posteriors of the MCMC for the base case, are more tightly clustered and suggest sustainable stock status, as F is around *F*_*MSY*_ and there is only a small probability that the stock falls below *B*_*MSY*_. In contrast, the yellow points of the uncertainty grid representing deterministic model estimates show a wide dispersion and a high uncertainty about the current status.

**Fig 10:**
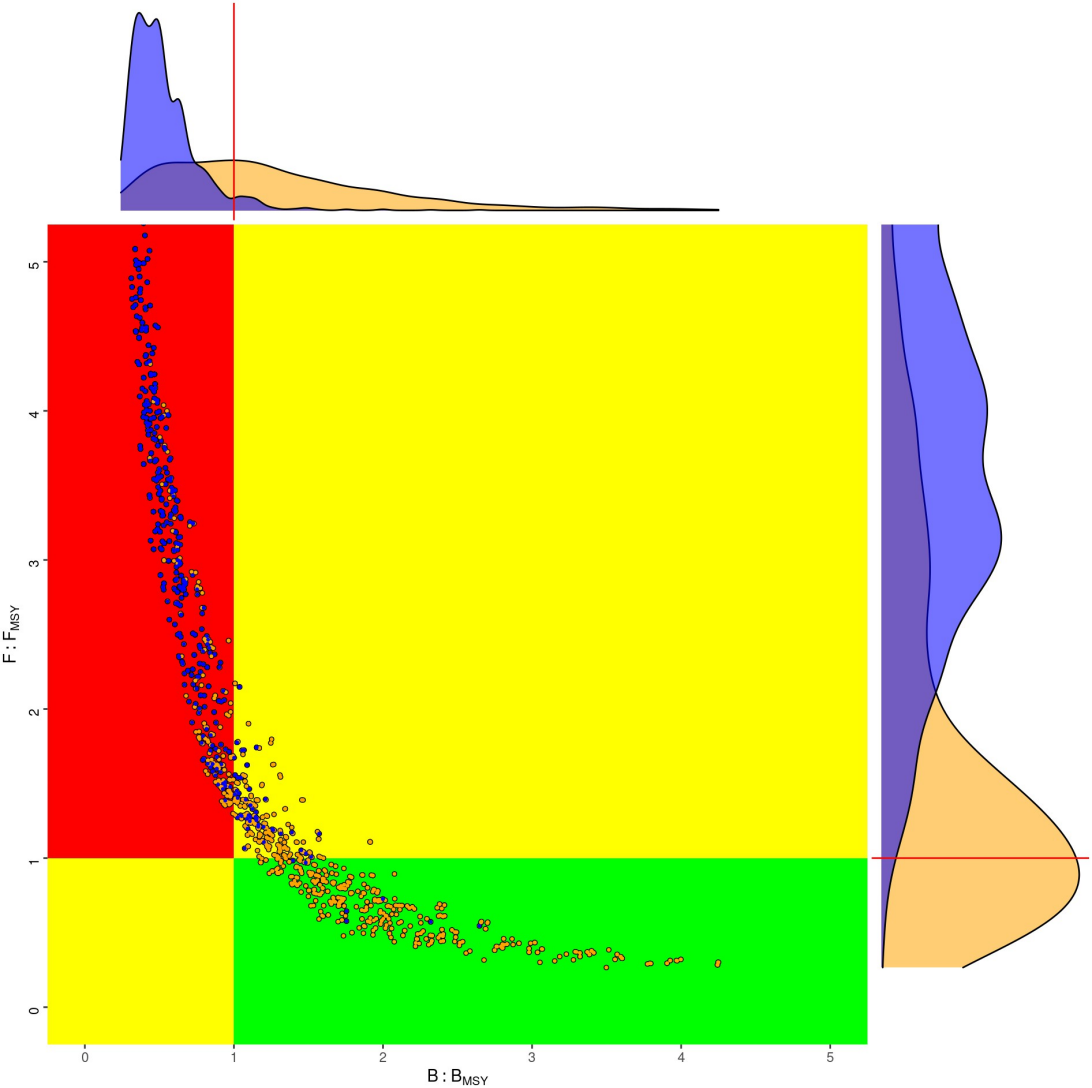
Kobe phase plots for uncertainty grid, where the equally weighted assessments (yellow) are compared to the scenarios which pass the Mohn’s *ρ* test.

**Fig 11:**
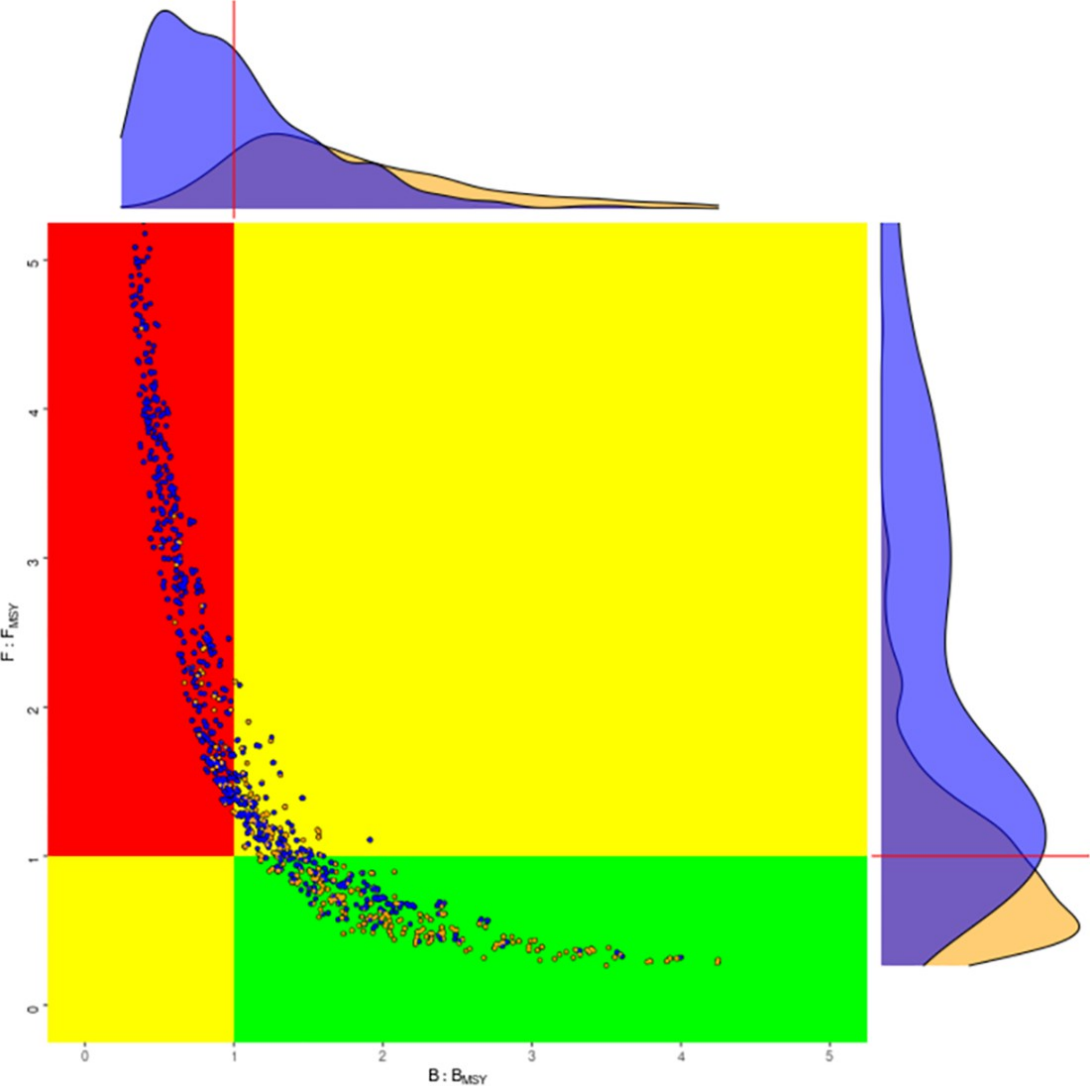
Kobe phase plots for uncertainty grid, where the equally weighted assessments (yellow) are compared to scenarios chosen based on prediction skill, i.e. MASE<1.

Variability in the predictions of the deterministic models implies that management advice is actually more uncertain than if the base case or a best assessment and MCMC had been used to provide advice. Since Figs 10 and 11 show that depending on the choice of scenarios, the stock is being fished sustainably or unsustainablely and may result in conscious or unconcious bias. This shows the importance of having a pre-greed procedure for selecting, rejecting, and weighting scenarios.

Although 1440 scenarios were evaluated, the uncertainty mainly affected the shape of the production function, scale, and level of process error. The status of the stock relative to the reference points was primarily influenced by fixed variables, e.g., adult M and steepness, while absolute biomass was affected by the relative importance of the CPUE and length data and the level of process error by adult M. There was confounding between the effects of steepness and natural mortality, both of which are crucial in determining sustainability, since higher natural mortality and steepness are associated with a steep slope at the origin and a skewed production function. The production function in turn determines the reference points for fishing mortality and how far below *B*_*MSY*_ or virgin biomass a stock can fall below productivity is impaired. The sensitivity of the assessment outputs to critical but commonly fixed biological factors such as natural mortality and steepness underscores the limitations of the best assessment if the fixed inputs do not fully capture the range of uncertainties inherent in fish stock assessments.

### Plausibility

The initial choice of scenarios and subsequent rejection, acceptance, and weighting is important in determining the status of the stock and subsequent management action. However, estimates from an ensemble may be biased if the models are a subset of all plausible models, some are less likely than others, or the models are non-unique causing redundancy [57]. Therefore, assuming the same reliability to all models could introduce bias, so ideally, each model should be assigned a weight [58] based on plausibility. Although the importance of plausibility is widely acknowledged, it is rarely formally defined. This lack of formal definition poses challenges in assessing the credibility and reliability of modelling results, potentially undermining the effectiveness of management strategies. Plausibility refers to the quality of seeming reasonable or probable based on the available evidence and logical coherence. A good example of the value of using observations not used in model fitting is that of [59], where a model was rejected based on alternative data that were not used in the assessment model, in this case fisher-derived data showing that one model was implausible. In another case in the ICCAT bluefin assessment conducted using virtual population analysis in which numbers alone are used, the predictions of adult biomass were inconsistent with observations of mean size of older individuals that identified model misspecification [60].

### Fisheries management advice

Managing fisheries poses challenges due to uncertainties and risks that arise from natural variability, imperfect information on aquatic ecosystems, and the inability to fully control fisheries [61]. Therefore, a stock assessment is performed to provide probabilistic statements about the status of stocks and their response to management. The risk of stock depletion or failure to consistently achieve objectives should be equivalent across all data quality categories and assessment methods [62]. The consideration of risk equivalence allows for a formal treatment of uncertainty so that management decisions can deliver consistent results [8] as required in tiered assessment frameworks and move toward an ecosystem approach to fishing (EAF).

However, estimating probabilities in stock assessments is difficult and requires a comprehensive approach to incorporate uncertainties and associated risks. Uncertainty sources include parameters for which there is minimal information in the data, model structure, and process variability. Bayesian MCMC methods can handle parameter uncertainty, and reversible jump MCMC methods model structure [63]. However, computational demands and the potential for misspecification of MCMC limit its application in the time frame of stock assessment working groups. Instead, assessment groups often use scenario testing as a robustness check, but they may then combine scenarios to provide advice [19], thus confusing sensitivity and uncertainty analysis.

The choice between sensitivity analysis and uncertainty analysis depends on the objectives of the assessment and the nature of the available data. Sensitivity analysis is beneficial to prioritise research efforts, while uncertainty analysis is crucial for a comprehensive understanding of potential outcomes. An objective approach for selecting, screening, and weighting hypotheses should be preagreed, to avoid “cherry picking”. The validation of the model should be performed using a diagnostic toolbox [10] with focus on prediction skill [64]. Prediction skill can be used to help identify and test alternative hypotheses to compare different modelling frameworks, to explore model misspecification and data conflicts, and to weight scenarios.

Once a sensitivity analysis has been performed, it can be used to determine whether estimates from alternative models fall outside the confidence or credibility intervals of a reference case. If the estimation error is less than the model error, this can indicate a lack of information in the data. The estimation error is related to the type and quality of the data, the estimable parameters and the variance assumed for priors and observations. Therefore, a high estimation error can indicate a lack of contrast in the data or a violation of the assumptions of the model. If the model error is greater than the estimation error, then statements about achieving targets and avoiding limits are model-dependent, and so an ensemble of models should be built.

However, conducting a full uncertainty analysis is difficult, especially when there is a great uncertainty. Therefore, sensitivity analysis is generally preferred to identify factors that are of high risk. For example, as in this case, it was found that the natural mortality of juveniles had little impact, but that of adults had a large effect on the production function, reference points, and the level of process error. The weight given to the length data determined the scale, that is, the absolute biomass and MSY. A sensitivity analysis can be used to agree Operating Models for the evaluation of management strategies to evaluate robust management strategies. However, after the implementation of an agreed management strategy, performance must be reviewed. This should be done less frequently than stock assessments used to set catch limits and, if possible, a comprehensive assessment conducted using an uncertainty analysis. This will provide an opportunity to learn from the implementation and apply lessons learnt in future iterations of the management cycle. By continuously refining strategies based on empirical evidence and practical experience, the management process becomes more dynamic and responsive to changing conditions and new information helping in moving towards EAF.

In MSE conditioning Operating Models in the form of an uncertainty grid, and then integrating outcomes to derive probabilities for performance metrics might obscure critical risk assessments. For example, the risk of falling below acceptable limits is commonly found from the tails of probability distributions. They may be captured more usefully through scenarios, i.e. a subset of Operating Models that embody specific uncertainties, and concerns of stakeholders. In this study, we showed that despite 1440 scenarios, there were 3 main outcomes; the shape of the production function, the scale, and the level of process error. An empirical management procedure could be tuned to provide robust advice on a much reduced subset of Operating Models. Therefore, our results support the use of sensitivity rather than uncertainty analysis for conditioning Operating Models, which examines the effects of varying key parameters and model structures. Therefore, provide a clearer identification of scenarios that significantly influence management outcomes. This helps to achieve the pragmatic goals of MSE by identifying robust management strategies and research priorities. To ensure that management advice is comprehensive and resilient, we advocate for a more deliberate and scenario-focused analysis to inform fisheries management decisions.

### Model development

Developing and validating models is crucial to address the complexities and uncertainties in fisheries management. Therefore, we propose a systematic and transparent approach to support a robust decision based on the following stages.

#### Identifying key uncertainties

The process begins by identifying critical uncertainties that affect stock assessments, including data quality, model structure, parameter estimation, and variability in fish population and fishing. Integrating stakeholder concerns is also increasingly vital as we transition to an EAF, ensuring that the models reflect the varied values and priorities within the fisheries system. For example, eliciting concerns, preferences, and objectives through interviews, workshops, and surveys enables the inclusion of various perspectives, such as those of fishermen, conservationists, industry representatives, and indigenous communities [65]. This approach promotes a transparent and inclusive fisheries management process that leads to sustainable and equitable results. Stakeholder participation during model development helps build ownership and trust, crucial for effective implementation of advice.

#### Selection of model candidates

Once uncertainties have been identified, a diverse suite of model candidates can be proposed, where the model may represent different hypotheses regarding stock dynamics, but also variations in life history traits, fishing pressure, environmental impacts, and ecosystem interactions. The selection depends on the focus, for example, whether it is on a single species or an ecosystem approach as we transition toward more integrated fisheries management practices.

#### Development of individual models

The next stage is the development and independent diagnostic evaluation of individual models. This involves structuring the model, fixing parameters or agreeing priors, and then fitting the model to the available data.

#### Weighting and integration of models

*Algorithm*. Therefore, we propose the following algorithm to develop stock assessment models used to provide and review the implementation of advice.

1. Run pre-agreed diagnostics to develop a reference case
2. Develop scenarios for plausible hypotheses that have a large effect
3. if model error is less than estimation error, then you can use the reference case for advice (i.e. best assessment paradigm)
4. If model error is greater than estimation error agree on scenarios, then an ensemble may better reflect uncertainty (i.e. ensemble paradigm) and the associated risks and should be preferred over a best-case scenario. This will be context-sensitive, but the rationale should be clearly stated
5. Fit scenarios and repeat diagnostics
6. Weight model scenarios in the ensemble based on diagnostics

A way forward is to use a discrete weight system (*W*(*D*)) based on diagnostic scores [66] to provide an estimate of plausibility based on the fit to the data.

The components *W*(*D*) can be calculated based on a series of interconnected diagnostic tests [10].

$$\begin{array}{c} \frac{W\left(D_{1}\right)+W\left(D_{1}\right)\ldots +W\left(D_{1}\right)}{\sum_{i=1}^{n}W(D_{i})} \end{array}$$ (3)

Each component *W* is assigned a value of 1 when the run passes the diagnostic test and a 0 if it fails. In addition, different weights could be assigned for the different diagnostic tests used. This provides an extension of current practice, and as more research is conducted on model weighting, this can be adapted.

*Management advice*. Models should be validated against independent data to evaluate the robustness and reliability of their outputs as part of an iterative process of development and refinement to maintain the relevance and accuracy of the models as new data emerge and our understanding of ecosystems evolves. This allows decision makers to account for uncertainties and risks. This structured approach, by continuously integrating and comparing various models, supports adaptive fisheries management.

## Conclusions

The three paradigms of best assessment, model ensemble, and MSE are all critical to adopting a precautionary approach to fisheries management, as they require the quantification of uncertainties and the associated risks that may impede the achievement of management goals. Statements of plausibility need to be supplemented by judgments of imprecise probability and knowledge strength. That an event or scenario is plausible is a vague statement and a scientific approach requires precision on both likelihood and knowledge [67]. Model validation, using prediction skill, provides a rigorous assessment of the plausibility of models and scenarios, using empirical evidence. Thus ensuring that advice is both credible and reliable. While, ensemble models, which incorporate an extensive array of plausible scenarios, will enhance our ability to encapsulate the inherent variability and uncertainty characteristic of fish stock assessments.

Moving toward an EAF requires continuous refinement and adjustments in response to evolving data and emerging insights, ensuring that management strategies take into account changing conditions and incorporate the latest evidence, thus improving their effectiveness. The incorporation of prediction skill as a metric for validation across all paradigms will improve the robustness of the management advice provided. Prediction skill provides an objective framework for the selection, rejection, and weighting of models or Operating Models ensuring that they are based on plausible hypotheses. By adhering to the principles of risk equivalence and embracing model validation through prediction skill, fisheries management can develop strategies that are not only resilient and sustainable, but also informed by a deep understanding of ecosystem dynamics and supported by empirical validation.

## Supporting information

S1 Appendix Case study and methodological details. (PDF)

## References

[1] Kell LT, Sharma R, Kitakado T, Winker H, Mosqueira I, Cardinale M, et al. Validation of stock assessment methods: is it me or my model talking? ICES Journal of Marine Science. 2021;78(6):2244–2255. doi: 10.1093/icesjms/fsab104

[2] Eker S, Rovenskaya E, Obersteiner M, Langan S. Practice and perspectives in the validation of resource management models. Nature communications. 2018;9(1):1–10. doi: 10.1038/s41467-018-07811-9 30560876 PMC6299083

[3] Saltelli A, Mayo D, Pielke R Jr, Portaluri T, Porter T, Puy A, et al. Five ways to ensure that models serve society: a manifesto. Nature. 2020;582 (7813). doi: 10.1038/d41586-020-01812-9 32581374

[4] Thygesen UH, Albertsen CM, Berg CW, Kristensen K, Nielsen A. Validation of ecological state space models using the Laplace approximation. Environmental and Ecological Statistics. 2017;24(2):317–339. doi: 10.1007/s10651-017-0372-4

[5] Kell LT, Kimoto A, Kitakado T. Evaluation of the prediction skill of stock assessment using hindcasting. Fisheries research. 2016;183:119–127. doi: 10.1016/j.fishres.2016.05.017

[6] Weigel A, Liniger M, Appenzeller C. Can multi-model combination really enhance the prediction skill of probabilistic ensemble forecasts? Quarterly Journal of the Royal Meteorological Society. 2008;134(630):241–260. doi: 10.1002/qj.210

[7] Garcia S. The precautionary approach to fisheries and its implications for fishery research, technology and management: an updated review. FAO Fisheries Technical Paper. 1996; p. 1–76.

[8] Roux MJ, Duplisea DE, Hunter KL, Rice J. Consistent risk management in a changing world: risk equivalence in fisheries and other human activities affecting marine resources and ecosystems. Frontiers in Climate. 2022;3:781559. doi: 10.3389/fclim.2021.781559

[9] Fischer SH, De Oliveira JA, Mumford JD, Kell LT. Risk equivalence in data-limited and data-rich fisheries management: An example based on the ICES advice framework. Fish and Fisheries. 2023;24(2):231–247. doi: 10.1111/faf.12722

[10] Carvalho F, Winker H, Courtney D, Kapur M, Kell L, Cardinale M, et al. A Cookbook for Using Model Diagnostics in Integrated Stock Assessments. Fisheries Research. 2021;240:105959. doi: 10.1016/j.fishres.2021.105959

[11] Lee HH, Maunder MN, Piner KR, Methot RD. Estimating natural mortality within a fisheries stock assessment model: an evaluation using simulation analysis based on twelve stock assessments. Fish Res. 2011;109(1):89–94. doi: 10.1016/j.fishres.2011.01.021

[12] Lee HH, Maunder MN, Piner KR, Methot RD. Can steepness of the stock–recruitment relationship be estimated in fishery stock assessment models? Fish Res. 2012;125:254–261. doi: 10.1016/j.fishres.2012.03.001

[13] Jiao Y, Smith EP, O’Reilly R, Orth DJ. Modelling non-stationary natural mortality in catch-at-age models. ICES Journal of Marine Science. 2012;69(1):105–118. doi: 10.1093/icesjms/fsr184

[14] Simon M, Fromentin JM, Bonhommeau S, Gaertner D, Brodziak J, Etienne MP. Effects of stochasticity in early life history on steepness and population growth rate estimates: An illustration on Atlantic bluefin tuna. PloS one. 2012;7(10):e48583. doi: 10.1371/journal.pone.0048583 23119063 PMC3485314

[15] Mourato B, Winker H, Carvalho F, Ortiz M. Stock Assessment of Atlantic blue marlin (Makaira nigricans) using a Bayesian State-Space Surplus Production Model JABBA. Collect Vol Sci Pap ICCAT. 2018;75(5):1003–1025.

[16] Neubauer P, Large K, Brouwer S. Stock assessment of Southwest Pacific blue shark. SCIENTIFIC COMMITTEE SEVENTEENTH REGULAR SESSION ELECTRONIC MEETING. 2021;WCPFC-SC17-2021/SA-WP-03.

[17] Punt A. The comparative performance of production-model and ad hoc tuned VPA based feedback-control management procedures for the stock of Cape hake off the west coast of South Africa. Canadian Special Publication of Fisheries and Aquatic Sciences. 1993; p. 283–300.

[18] Kell L, De Oliveira JA, Punt AE, McAllister MK, Kuikka S. Operational management procedures: an introduction to the use of evaluation frameworks. Developments in Aquaculture and Fisheries Science. 2006;36:379–407. doi: 10.1016/S0167-9309(06)80018-9

[19] Kell LT, Levontin P, Davies CR, Harley S, Kolody DS, Maunder MN, et al. The quantification and presentation of risk. Management Science in Fisheries: An Introduction to Simulation-based Methods. 2016; p. 348.

[20] Polacheck T, Klaer N, Millar C, Preece A. An initial evaluation of management strategies for the southern bluefin tuna fishery. ICES Journal of Marine Science. 1999;56(6):811–826. doi: 10.1006/jmsc.1999.0554

[21] Loucks DP, Van Beek E. Water resource systems planning and management: An introduction to methods, models, and applications. Springer; 2017.

[22] Pechlivanidis I, Gupta H, Bosshard T. An Information Theory Approach to Identifying a Representative Subset of Hydro-Climatic Simulations for Impact Modeling Studies. Water resources research. 2018;54(8):5422–5435. doi: 10.1029/2017WR022035 30344354 PMC6175403

[23] Fromentin JM, Bonhommeau S, Arrizabalaga H, Kell L LT. The spectre of uncertainty in management of exploited fish stocks: the illustrative case of Atlantic Bluefin tuna. Marine Policy. 2014;47:8–14. doi: 10.1016/j.marpol.2014.01.018

[24] Rajabi MM, Fahs M, Panjehfouladgaran A, Ataie-Ashtiani B, Simmons CT, Belfort B. Uncertainty quantification and global sensitivity analysis of double-diffusive natural convection in a porous enclosure. International Journal of Heat and Mass Transfer. 2020;162:120291. doi: 10.1016/j.ijheatmasstransfer.2020.120291

[25] Jardim E, Azevedo M, Brodziak J, Brooks EN, Johnson KF, Klibansky N, et al. Operationalizing ensemble models for scientific advice to fisheries management. ICES Journal of Marine Science. 2021;78(4):1209–1216. doi: 10.1093/icesjms/fsab010

[26] Walker WE, Marchau VA, Kwakkel JH. Uncertainty in the framework of policy analysis. In: Public policy analysis. Springer; 2013. p. 215–261.

[27] Decker C. Utility and regulatory decision-making under conditions of uncertainty: Balancing resilience and affordability. Utilities Policy. 2018;51:51–60. doi: 10.1016/j.jup.2018.01.007

[28] Anderson SC, Cooper AB, Jensen OP, Minto C, Thorson JT, Walsh JC, et al. Improving estimates of population status and trend with superensemble models. Fish and Fisheries. 2017;18(4):732–741. doi: 10.1111/faf.12200

[29] Langley A, Hoyle S. Stock assessment of albacore tuna in the Indian Ocean using Stock Synthesis. IOTC WPTmT. 2016;IOTC-2016-WPTmT06-25.

[30] Hoyle S, Fu D, Kim DN, Lee SI, Matsumoto T, Satoh K, et al. Collaborative study of albacore tuna CPUE from multiple Indian Ocean longline fleets in 2019. IOTC WPTm07(AS), Shizuoka (JP), 23–27 July 2019. 2016;IOTC-2019-WPTmT07(AS)-10.

[31] Kolody D, Polacheck T, Basson M, Davies C. Salvaged pearls: lessons learned from a floundering attempt to develop a management procedure for Southern Bluefin Tuna. Fisheries Research. 2008;94(3):339–350. doi: 10.1016/j.fishres.2008.08.016

[32] Sharma R, Levontin P, Kitakado T, Kell L, Mosqueira I, Kimoto A, et al. Operating model design in tuna Regional Fishery Management Organizations: Current practice, issues and implications. Fish and Fisheries. 2020;21(5):940–961. doi: 10.1111/faf.12480

[33] Kurota H, Hiramatsu K, Takahashi N, Shono H, Itoh T, Tsuji S. Developing a management procedure robust to uncertainty for southern bluefin tuna: a somewhat frustrating struggle to bridge the gap between ideals and reality. Population Ecology. 2010;52(3):359–372. doi: 10.1007/s10144-010-0201-1

[34] Merino G, Murua H, Santiago J, Arrizabalaga H, Restrepo V. Characterization, Communication, and Management of Uncertainty in Tuna Fisheries. Sustainability. 2020;12(19):8245. doi: 10.3390/su12198245

[35] Tremblay-Boyer L, Hampton J, McKechnie S, Pilling G. Stock assessment of South Pacific albacore tuna. 14th Regular Session of the Scientific Committee of the WCPFC Busan, Republic of Korea. 2018;.

[36] McKechnie S, Hampton J, Pilling G, Davies N. Stock assessment of skipjack tuna in the western and central Pacific Ocean. Scientific Committee twelfth Regular Session Bali, Indonesia. 2016; p. 3–11.

[37] IOTC. Report of the 7th Session of the IOTC Working Party on Temperate Tunas: Assessment Meeting. Shizuoka, Japan: Indian Ocean Tuna Commission; 2019. IOTC-2019-WPTmT07(AS)-R.

[38] NEAL R. Probabilistic inference using Markov chain monte carlo methods. Technical Report CRGTR-93-1. 1993;.

[39] IOTC. Report of the Sixth Session of the IOTC Working Party on Temperate Tunas. IOTC WPTmT06, Shanghai (CH), 18–21 July 2016. 2014;IOTC-2016-WPTmT06-R[E].

[40] Methot RD, Wetzel CR. Stock synthesis: a biological and statistical framework for fish stock assessment and fishery management. Fisheries Research. 2013;142:86–99. doi: 10.1016/j.fishres.2012.10.012

[41] Secretariat I. Review of the statistical data and fishery trends for albacore. IOTC WPTmT06, Shanghai (CN) 18-21 July 2016. 2016;IOTC-2016-WPTmT06-07.

[42] Rosenberg AA, Restrepo VR, et al. Precautionary management reference points and management strategies. FAO Fisheries Technical Paper. 1996; p. 129–140.

[43] van den Berg NI, Machado D, Santos S, Rocha I, Chacón J, Harcombe W, et al. Ecological modelling approaches for predicting emergent properties in microbial communities. Nature Ecology & Evolution. 2022; p. 1–11. doi: 10.1038/s41559-022-01746-7 35577982 PMC7613029

[44] Sissenwine MP, Shepherd JG. An alternative perspective on recruitment overfishing and biological reference points. Canadian Journal of Fisheries and Aquatic Sciences. 1987;44(4):913–918. doi: 10.1139/f87-110

[45] Maunder MN, Piner KR. Contemporary fisheries stock assessment: many issues still remain. ICES Journal of Marine Science. 2014;72(1):7–18. doi: 10.1093/icesjms/fsu015

[46] Ahrestani FS, Hebblewhite M, Post E. The importance of observation versus process error in analyses of global ungulate populations. Scientific reports. 2013;3(1):1–10. doi: 10.1038/srep03125 24201239 PMC6506149

[47] Punt AE. Those who fail to learn from history are condemned to repeat it: A perspective on current stock assessment good practices and the consequences of not following them. Fisheries Research. 2023;261:106642. doi: 10.1016/j.fishres.2023.106642

[48] Walters CJ, Hilborn R, Christensen V. Surplus production dynamics in declining and recovering fish populations. Canadian Journal of Fisheries and Aquatic Sciences. 2008;65(11):2536–2551. doi: 10.1139/F08-170

[49] Wald A, Wolfowitz J. On a test whether two samples are from the same population. The Annals of Mathematical Statistics. 1940;11(2):147–162. doi: 10.1214/aoms/1177731909

[50] Sugiura N. Further analysts of the data by akaike’s information criterion and the finite corrections: Further analysts of the data by akaike’s. Communications in Statistics-Theory and Methods. 1978;7(1):13–26. doi: 10.1080/03610927808827599

[51] Konishi S, Kitagawa G. Information criteria and statistical modeling. Springer Science & Business Media; 2008.

[52] Spiegelhalter DJ, Best NG, Carlin BP, Van Der Linde A. Bayesian measures of model complexity and fit. Journal of the royal statistical society: Series b (statistical methodology). 2002;64(4):583–639. doi: 10.1111/1467-9868.00353

[53] Watanabe S. Asymptotic equivalence of Bayes cross validation and widely applicable information criterion in singular learning theory. Journal of machine learning research. 2010;11:3571–3594.

[54] Mohn R. The retrospective problem in sequential population analysis: an investigation using cod fishery and simulated data. ICES Journal of Marine Science. 1999;56(4):473–488. doi: 10.1006/jmsc.1999.0481

[55] Hyndman RJ, Koehler AB. Another look at measures of forecast accuracy. International Journal of Forecasting. 2006;22(4):679–688. doi: 10.1016/j.ijforecast.2006.03.001

[56] Diebold FX, Mariano RS. Comparing predictive accuracy. Journal of Business & economic statistics. 2002;20(1):134–144. doi: 10.1198/073500102753410444

[57] Dormann CF, Calabrese JM, Guillera-Arroita G, Matechou E, Bahn V, Bartoń K, et al. Model averaging in ecology: A review of Bayesian, information-theoretic, and tactical approaches for predictive inference. Ecological Monographs. 2018;88(4):485–504. doi: 10.1002/ecm.1309

[58] Francis RC. Data weighting in statistical fisheries stock assessment models. Canadian Journal of Fisheries and Aquatic Sciences. 2011;68(6):1124–1138. doi: 10.1139/f2011-025

[59] Duplisea DE. Eliminating implausible fisheries assessment models using fishers’ knowledge. Canadian Journal of Fisheries and Aquatic Sciences. 2018;75(8):1280–1290. doi: 10.1139/cjfas-2017-0178

[60] International Commission for the Conservation of Atlantic Tunas. Report of the 2017 ICCAT Bluefin Stock Assessment Meeting (Madrid, Spain—July 20 to 28, 2017); 2017.

[61] Peterman RM. Possible solutions to some challenges facing fisheries scientists and managers. ICES Journal of Marine Science. 2004;61(8):1331–1343. doi: 10.1016/j.icesjms.2004.08.017

[62] Fulton EA, Punt AE, Dichmont CM, Gorton R, Sporcic M, Dowling N, et al. Developing risk equivalent data-rich and data-limited harvest strategies. Fisheries Research. 2016;183:574–587. doi: 10.1016/j.fishres.2016.07.004

[63] Green PJ. Reversible jump Markov chain Monte Carlo computation and Bayesian model determination. Biometrika. 1995;82(4):711–732. doi: 10.1093/biomet/82.4.711

[64] Hodges JS, Dewar JA, Center A. Is it you or your model talking?: A framework for model validation. Santa Monica, CA: Rand; 1992.

[65] Leach AW, Levontin P, Holt J, Kell LT, Mumford JD. Identification and prioritization of uncertainties for management of Eastern Atlantic bluefin tuna (Thunnus thynnus). Marine Policy. 2014;48:84–92. doi: 10.1016/j.marpol.2014.03.010

[66] Maunder MN, Xu H, Lennert-Cody CE, Valero JL, Aires-da Silva A, Minte-Vera C. Implementing reference point-based fishery harvest control rules within a probabilistic framework that considers multiple hypotheses (No. SAC-11-INF-F). Scientific Advisory Commitee, Inter-American Tropical Tuna Commission, San Diego. 2020;.

[67] Glette-Iversen Ingrid, Aven Terje, & Flage Roger. 2022. The concept of plausibility in a risk analysis context: Review and clarifications of defining ideas and interpretations. *Safety Science*, 147, 105635. doi: 10.1016/j.ssci.2021.105635

