# Supplementary material for: Empirical validation of integrated stock assessment models to ensuring risk equivalence: A pathway to resilient fisheries management
Source: PLoS One. 2024 Jul 2;19(7):e0302576. doi: 10.1371/journal.pone.0302576 (PMC11218941; doi:10.1371/journal.pone.0302576)
Supplement: S1 Appendix — (PDF) [file pone.0302576.s001.pdf]

Appendix S1. Case study and methodological details.

Model Consistency

Model consistency was evaluated based on retrospective analysis using relative error (RE) to identify systematic patterns across assessment periods. In retrospective analysis all observations for a year are sequentially removed from the terminal year backwards (i.e. peeled), the model is then refitted to the truncated series and model estimates are compared using Mohn's ρ [1]. We use a variant scaled by the mean, so the metric is not affected by the peel's length or the number of steps ahead.

$$\rho_M = \frac{1}{n} \sum_{t=T-n}^{T-1} \frac{\hat{y}_{(1:t),t} - \hat{y}_{(1:T),t}}{\hat{y}_{(1:T),t}} \tag{1}$$

where n is the number of time steps that the peel is performed for, t is the time for which the missing value is being estimated, T is the terminal year in the time series, and $\hat{y}$ denotes a model-based quantity. The value with suffix $\hat{y}_{(1:T),t}$ means a value estimated at a time t from the full series running from time 1 to T, and $\hat{y}_{(1:t),t}$ is the value estimated using the data window from 1 to $t(\leq T)$. ρ_M is an average of the relative differences at the final time of each window and is a measure of relative retrospective 'bias' (scale-free) in a statistical sense. The metric tends to be applied not on the log but on the original scale because both positive and negative directions are equivalent.

Here, we compute retrospective bias [2] ρ estimated for one year and three years of forecast horizons, i.e. h-steps ahead.

$$\rho_M = \frac{1}{n-h+1} \sum_{t=T-n}^{T-h} \frac{\hat{y}_{(1:t),t+h} - \hat{y}_{(1:T),t+h}}{\hat{y}_{(1:T),t+h}} \tag{2}$$

There is no upper limit for reference values that are low relative to the alternative, while in the reverse case, the error cannot exceed 1.0. Therefore, in practice, it is usual to use a lower bound of -0.15 and an upper bound of 0.20 to identify acceptable performance for long-lived species [3]. For values near or equal to 0, e.g. stocks where exploitation or stock size is low, small absolute differences in the abundance of fishing pressure can result in large relative differences. This may result in assessments being rejected when needed the most, e.g. during the development of recovery plans, when both stock biomass and fishing mortality may be low.

Model Validation

Validation requires that the system is observable and measurable, and so observations should be used [4], rather than model-based quantities unless these are well known. Hindcasting is a way of validating models that use different data sets and penalty terms based on the skill of models in predicting future observations. The models were used to generate pseudo-data based on CPUE which were then compared to the actual observations. In the procedure used in this study, only CPUE observations were removed, while catch and length composition remained in the model because the composition data were not provided by the fleet (i.e. CPUE index). This was because these data had been combined due to the low level of sampling. Due to difficulties with collecting composition data from fisheries from areas beyond national jurisdiction, a

single set of composition data based on all samples had been provided by IOTC for the stock assessment.

Like retrospective analysis, hindcasting involves fitting a model using a tail-cutting procedure, where data are deleted sequentially. This may be done for individual data series or combinations of different series, for example, fleets where both CPUE and length data are removed. This allows data conflicts to be explored. Theoretically, the projection period is to the end of the historical period [2]. However, in practice, a step size of one or several years ahead (the horizon h) is chosen for cross-validation against observations removed from the model fit. This reflects the time horizon required for robust management advice, considering the typically high process stochasticity in fishery population dynamics and non-trivial extents of observation uncertainty. The objectives of stock assessments are not just to provide historical or current estimates of stock size but also to make predictions of future status, therefore a horizon of three, as well as one year, was also considered since assessment cycles are typically not performed annually but every three years in most Tuna Regional Fisheries Management Organisations.

Therefore, the implemented procedure is similar to a jackknife in that we remove points using a tail-cut (peel) and then "predict" missing values as part of the fitting process. Time series of pseudo-data were generated from estimates of vulnerable biomass and catchability (q). Prediction residuals (e) were then computed as the difference between the predictions and the observations [5]. It is also possible to perform hindcasting using other data, e.g.length or age compositions [6].

Prediction skill based on a forecast compares an observation at a time t (y_t) to a prediction of that observation made h time steps previously ($\hat{y}_{t|t-h}$). To compare forecasts we used the Mean Absolute Scaled Error (MASE), as it is a robust and easy-to-interpret statistic [7], and compares the prediction error (e_t) for a prediction horizon of h with the observations (Y_t).

$$e_t = y_t - \hat{y}_{t|t-h} \tag{3}$$

$$\text{MASE} = \text{mean}\left(\frac{|e_j|}{\frac{1}{T-1}\sum_{t=2}^{T}|Y_t - Y_{t-1}|}\right) = \frac{\frac{1}{J}\sum_j |e_j|}{\frac{1}{T-1}\sum_{t=2}^{T}|Y_t - Y_{t-1}|} \tag{4}$$

$$e_t = y_t - \hat{y}_{t|t-1} \tag{5}$$

$$\text{MASE} = \frac{\frac{1}{n}\sum_{t=T-n+1}^{T}|e_t|}{\frac{1}{n}\sum_{t=T-n+1}^{T}|y_t - y_{t-1}|} \tag{6}$$

to a benchmark forecast corresponding to a naïve forecast equal to the last observed value.

$$\hat{y}_{t|t-h} = y_{t-h} \tag{7}$$

For a peel of n and a horizon of h years

$$\text{MASE} = \frac{\frac{1}{n+1}\sum_{t=T-n}^{T}\left|y_t - \hat{y}_{t|t-h}\right|}{\frac{1}{n+1+h}\sum_{t=T-n-h}^{T}\left|y_t - y_{t-h}\ \right|} \tag{8}$$

The MASE has the desirable properties of scale invariance, so it can be used to compare forecasts across data sets with different scales, predictable behaviour, symmetry, interpretability and asymptotic normality. Unlike relative error, MASE does not skew its distribution even when the observed values are close to zero and is easy to interpret, as a score of 0.5 indicates that the model forecasts are twice as accurate as a

naïve baseline prediction. A value of MASE larger than 1 indicates that a random walk is better than the model prediction, so values less than 1 indicate a good model prediction skill.

The Diebold-Mariano test [8] for forecasts was used to test the statistical significance of the difference between two sets of forecasts, i.e. by comparing the prediction $y_t - \hat{y}_t|t-h$ to a random walk $y_t - y_{t-h}$.

Case Study

Tuna make up nearly 30% of the total global trade in seafood.The global tuna market was worth approximately US $12 billion in 2018with annual sales of US $42 billion, while in the Asia-Pacific region alone, the tuna industry directly employs more than 6 million people.To help provide consistency of advice across Tuna Regional Fisheries Management Organisations, a common management advice framework, i.e. the Kobe Framework [9] has been agreed upon. This requires stock assessment results to be reported regarding the probabilities of maintaining the stock above B_{MSY} and fishing mortality below F_{MSY}. Therefore, advice on stock status is given relative to MSY reference points. The Tuna Regional Management Fisheries Organisations all use integrated models to perform stock assessments, using software such as Stock Synthesis [10] and MultifanCL [11].

The Tuna Regional Fisheries Management Organisations commonly develop uncertainty grids to condition models in integrated stock assessments to account for uncertainties in parameters that cannot be estimated from the data [12–18]. For example, by constructing multidimensional grids that consist of different plausible combinations of assumptions, fixed parameter values, and data sets. It is not always clear, however, whether this is intended as an uncertainty analysis or a sensitivity analysis. The choice of assessment model scenarios and ways of estimating uncertainty has an impact on the risk of exceeding the limit and missing target reference points. Therefore, to better understand the impact of uncertainty on stock assessment advice and the risk of failing to meet conservation and sustainability objectives, we used the uncertainty grid developed by the Indian Ocean Tuna Commission (IOTC) for albacore tuna (*Thunnus alalunga*), a full factorial design with 1,440 model configurations [19]. This is sufficient to provide contrast, but not too big to be unmanageable.

Estimation Error

We used Bayesian Markov Chain Monte Carlo (MCMC) methods [20]. MCMC is a class of algorithms for sampling from a probability distribution. It is generally used in integrated models for detecting issues with an estimation of parameters and to derive the probability distribution of the key-derived quantities. By constructing a Markov chain, it is possible to obtain a sample of the desired distribution by observing the chain after several steps. MCMC methods create samples from a possibly multidimensional continuous random variable with probability density proportional to a known function. These samples can be used to evaluate an integral over that variable, such as its expected value or variance. Practically, one or more sets of chains are generally developed, starting from a set of points arbitrarily chosen and sufficiently distant from each other. Then those are used to estimate the posterior distribution of the parameters of interest within the model. To quantify the estimation error of SSB/SSB_{MSY} and F/F_{MSY}, we performed an MCMC for the base case scenario using the Random Walk Metropolis method with 1,200,000 iterations across 3 chains, a burn-in period 100,000 and thinning each 200 iterations. We confirmed that the chains had converged using various convergence diagnostics [21, 22] as implemented in the coda package in R.

References

1. Mohn R. The retrospective problem in sequential population analysis: an investigation using cod fishery and simulated data. ICES Journal of Marine Science. 1999;56(4):473–488.

2. Brooks EN, Legault CM. Retrospective forecasting—evaluating performance of stock projections for New England groundfish stocks. Canadian Journal of Fisheries and Aquatic Sciences. 2016;73(6):935–950.

3. Hurtado-Ferro F, Szuwalski CS, Valero JL, Anderson SC, Cunningham CJ, Johnson KF, et al. Looking in the rear-view mirror: bias and retrospective patterns in integrated, age-structured stock assessment models. ICES Journal of Marine Science. 2015;72(1):99–110.

4. Kell LT, Sharma R, Kitakado T, Winker H, Mosqueira I, Cardinale M, et al. Validation of stock assessment methods: is it me or my model talking? ICES Journal of Marine Science. 2021;78(6):2244–2255. doi:10.1093/icesjms/fsab104.

5. Itakura F. Minimum prediction residual principle applied to speech recognition. IEEE Transactions on acoustics, speech, and signal processing. 1975;23(1):67–72.

6. Carvalho F, Winker H, Courtney D, Kapur M, Kell L, Cardinale M, et al. A Cookbook for Using Model Diagnostics in Integrated Stock Assessments. Fisheries Research. 2021;240:105959. doi:https://doi.org/10.1016/j.fishres.2021.105959.

7. Hyndman RJ, Koehler AB. Another look at measures of forecast accuracy. International Journal of Forecasting. 2006;22(4):679–688. doi:https://doi.org/10.1016/j.ijforecast.2006.03.001.

8. Diebold FX, Mariano RS. Comparing predictive accuracy. Journal of Business & economic statistics. 2002;20(1):134–144.

9. De Bruyn P, Murua H, Aranda M. The Precautionary approach to fisheries management: How this is taken into account by Tuna regional fisheries management organisations (RFMOs). Marine Policy. 2012;.

10. Methot RD, Wetzel CR. Stock synthesis: a biological and statistical framework for fish stock assessment and fishery management. Fisheries Research. 2013;142:86–99.

11. Fournier D, Archibald CP. A general theory for analyzing catch at age data. Canadian Journal of Fisheries and Aquatic Sciences. 1982;39(8):1195–1207.

12. Kell LT, Levontin P, Davies CR, Harley S, Kolody DS, Maunder MN, et al. The quantification and presentation of risk. Management Science in Fisheries: An Introduction to Simulation-based Methods. 2016; p. 348.

13. Kolody D, Polacheck T, Basson M, Davies C. Salvaged pearls: lessons learned from a floundering attempt to develop a management procedure for Southern Bluefin Tuna. Fisheries Research. 2008;94(3):339–350. doi:https://doi.org/10.1016/j.fishres.2008.08.016.

14. Sharma R, Levontin P, Kitakado T, Kell L, Mosqueira I, Kimoto A, et al. Operating model design in tuna Regional Fishery Management Organizations: Current practice, issues and implications. Fish and Fisheries. 2020;21(5):940–961.

15. Kurota H, Hiramatsu K, Takahashi N, Shono H, Itoh T, Tsuji S. Developing a management procedure robust to uncertainty for southern bluefin tuna: a somewhat frustrating struggle to bridge the gap between ideals and reality. Population Ecology. 2010;52(3):359–372.

16. Merino G, Murua H, Santiago J, Arrizabalaga H, Restrepo V. Characterization, Communication, and Management of Uncertainty in Tuna Fisheries. Sustainability. 2020;12(19):8245.

17. Tremblay-Boyer L, Hampton J, McKechnie S, Pilling G. Stock assessment of South Pacific albacore tuna. 14th Regular Session of the Scientific Committee of the WCPFC Busan, Republic of Korea. 2018;.

18. McKechnie S, Hampton J, Pilling G, Davies N. Stock assessment of skipjack tuna in the western and central Pacific Ocean. Scientific Committee twelfth Regular Session Bali, Indonesia. 2016; p. 3–11.

19. IOTC. Report of the 7th Session of the IOTC Working Party on Temperate Tunas: Assessment Meeting. Shizuoka, Japan: Indian Ocean Tuna Commission; 2019. IOTC-2019-WPTmT07(AS)-R.

20. NEAL R. Probabilistic inference using Markov chain monte carlo methods. Technical Report CRGTR-93-1. 1993;.

21. Heidelberger P, Welch PD. Simulation run length control in the presence of an initial transient. Operations Research. 1983;31(6):1109–1144.

22. Geweke J. Evaluating the accuracy of sampling-based approaches to the calculations of posterior moments. Bayesian statistics. 1992;4:641–649.
